# Supplementary material for: Sleep Duration, Sleep Quality and Physical Activity, but Not Sedentary Behaviour, Are Associated with Positive Mental Health in a Multi-Ethnic Asian Population: A Cross-Sectional Evaluation
Source: Int J Environ Res Public Health. 2020 Nov 16;17(22):8489. doi: 10.3390/ijerph17228489 (PMC7697582; doi:10.3390/ijerph17228489)
Supplement: Supplementary file 1 [file ijerph-17-08489-s001.pdf]

Table S1: Association of physical activity in work, transport and leisure domains in relation to Positive Mental Health domains

Physical activity [@]	Multivariable Model 1*				Multivariable Model 2[#]			
	β	95% CI		P trend	β	95% CI		P trend
		Lower	Upper			Lower	Upper	
**Work domain**								
Total Positive Mental Health	0.070	0.040	0.100	<0.001	0.070	0.030	0.120	0.001
General coping	0.090	0.040	0.130	<0.001	0.090	0.030	0.140	0.002
Emotional support	0.040	-0.020	0.090	0.167	0.070	0.010	0.120	0.025
Spirituality	0.070	-0.010	0.16	0.076	0.030	-0.070	0.140	0.544
Interpersonal skills	0.090	0.050	0.130	<0.001	0.090	0.040	0.130	<0.001
Personal growth and autonomy	0.090	0.050	0.130	<0.001	0.100	0.050	0.150	<0.001
Global affect	0.00	-0.040	0.040	0.941	0.020	-0.040	0.070	0.559
**Transport domain**								
Total Positive Mental Health	0.100	0.040	0.160	0.001	0.060	0.010	0.110	0.027
General coping	0.120	0.060	0.190	<0.001	0.100	0.030	0.180	0.007
Emotional support	0.080	0.010	0.150	0.033	0.040	-0.050	0.120	0.399
Spirituality	0.110	-0.030	0.250	0.112	0.070	-0.070	0.220	0.322
Interpersonal skills	0.100	0.030	0.160	0.003	0.040	-0.030	0.120	0.277
Personal growth and autonomy	0.120	0.050	0.190	0.001	0.050	-0.030	0.130	0.196
Global affect	0.080	0.000	0.160	0.046	0.060	-0.030	0.160	0.188
**Leisure domain**								
Total Positive Mental Health	0.210	0.070	0.350	0.003	0.160	-0.040	0.350	0.110
General coping	0.230	-0.040	0.510	0.095	0.090	-0.330	0.500	0.680
Emotional support	0.220	0.070	0.380	0.004	0.230	0.040	0.430	0.020
Spirituality	0.030	-0.260	0.320	0.838	0.060	-0.340	0.460	0.759
Interpersonal skills	0.230	0.090	0.370	0.001	0.210	0.040	0.380	0.015
Personal growth and autonomy	0.280	0.140	0.420	<0.001	0.230	0.060	0.410	0.010
Global affect	0.200	0.090	0.320	0.001	0.060	-0.100	0.220	0.460

[@] Metabolic Equivalent Tasks (METs) in hours per week derived from moderate or vigorous activity in respective domains of activity, estimates are expressed for 10 MET hours/week, *General linear regression models adjusted for: Age, Gender (Men, Women), Ethnicity (Chinese, Malay, Indian, Other), [#]General linear regression models adjusted for: Age, Gender (Men, Women), Ethnicity (Chinese, Malay, Indian, Other), Marital status (Never married, Married, Separated/Divorced/ Widowed), Education level (Primary and below, Secondary, Post-secondary, University), Employment status (Employed, Unemployed),

Body Mass Index (underweight, normal weight, overweight, obese), Any chronic physical condition (Yes, No), Alcohol consumption in past 12 months (Excessive, Non-excessive, None), Current smoking status (Daily, Occasional, Past, Never).

Table S2: Sensitivity analysis investigating the multivariable-adjusted association of sleep duration in relation with total positive mental health score excluding participants with fairly or very bad sleep quality (n=1707)

	**β**	**95% CI**		**P trend**
		**Lower**	**Upper**	
**Sleep duration (hours per night)**				0.012
≥8	0.143	-0.034	0.319	
7-<8	0.153	-0.009	0.314	
6-<7	-0.019	-0.188	0.150	
<6	Ref			
**With different domains of Positive Mental Health (per 1 hr/night)**				
General coping	0.039	-0.017	0.096	0.175
Emotional support	0.088	0.030	0.145	0.003
Spirituality	0.068	-0.025	0.161	0.149
Interpersonal skills	0.026	-0.021	0.073	0.285
Personal growth and autonomy	0.057	0.006	0.108	0.028
Global affect	0.088	0.036	0.140	0.001

General linear regression models adjusted for: Age, Gender (Men, Women), Ethnicity (Chinese, Malay, Indian, Other), Marital status (Never married, Married, Separated/Divorced/ Widowed), Education level (Primary and below, Secondary, Post-secondary, University), Employment status (Employed, Unemployed), Body Mass Index (underweight, normal weight, overweight, obese), Any chronic physical condition (Yes, No), Alcohol consumption in past 12 months (Excessive, Non-excessive, None), Current smoking status (Daily, Occasional, Past, Never).
